# Isokinetic quadriceps physiotherapy after knee surgery: a retrospective study

**DOI:** 10.3389/fresc.2024.1336847

**Published:** 2024-05-16

**Authors:** Siniša Nikolić, Borislav Obradović, Vanja Dimitrijević, Bojan Rašković, Dragana Dragičević-Cvjetković

**Affiliations:** ^1^ Institute of Physical Medicine, Rehabilitation and Orthopedic Surgery “Dr. Miroslav Zotović”, Banja Luka, Bosnia and Herzegovina; ^2^ Faculty of Medicine, Department of Physiotherapy, University of Banja Luka, Banja Luka, Bosnia and Herzegovina; ^3^ Faculty of Sport and Physical Education, University of Novi Sad, Novi Sad, Serbia; ^4^ Faculty of Medicine, Department of Physical Medicine and Rehabilitation, University of Banja Luka, Banja Luka, Bosnia and Herzegovina

**Keywords:** isokinetic, isotonic, exercise, knee surgery, biomechanics

## Abstract

**Introduction:**

Quadriceps weakness after knee surgery is the most common consequence that can have different consequences not only for the knee itself but also for the locomotor system in general. This study aimed to compare the results of isokinetic and isotonic exercise on torque restoration quadriceps on knee surgery.

**Methods:**

A sample of 180 subjects was analyzed and divided into two subsamples according to the type of rehabilitation protocol that was implemented. The examined group A-isokinetic consisted of 90 male subjects aged 28.54 ± 4.44 years, with a rehabilitation protocol based on the isokinetic exercise of the quadriceps. The examined group B-isotonic also consisted of 90 male subjects aged 27.93 ± 4.27 years, with a rehabilitation protocol for strengthening the quadriceps that applied an exercise program with additional resistance, i.e., isotonic exercise in the gym. Before the start of the rehabilitation treatment, an initial isokinetic test was performed at an angular speed of 60 °/s in all subjects. After 3 and 6 weeks of rehabilitation treatment, the control tests were performed in the same way as in the initial test.

**Results:**

Based on the values of MANOVA analysis and discriminative analysis, significantly better results of isokinetic tests were found in the examined group A-isokinetic compared with those in the examined group B-isotonic. At the final measurement of group A, 83 respondents (92.2%) were placed in the “biggest” class out of the 90 respondents.

**Conclusion:**

Based on the obtained research results, we conclude that isokinetic exercise is more effective in terms of physiotherapy of quadriceps hypotrophy after knee surgery.

## 1 Introduction

Exposure of the locomotor system to large external and internal forces during sports activities is one of the many causes of injuries, with the most common being knee injury. One of the most common injuries in sports and recreation is an injury to the anterior cruciate ligament (ACL) of the knee, resulting in a large number of cases of chronic osteoarthritis and permanent disability of the knee joint (1–3). They are especially reported in active athletes and recreational sportsmen in football, skiing, handball, or volleyball (4, 5), with women being exposed to a 2–9 times higher risk than men (6–10). Elite athletes can develop significant muscle asymmetry during daily training, and the specificity of sports movements can lead to muscle imbalance, potentially increasing the risk of injuries (11, 12). The incidence of acute ACL injuries in the USA accounted for 1:3,000 per year (13). A large number of studies have focused on the development of surgical techniques (14) rather than on the analysis of injury causes, risk factors, and injury prevention (6–10, 13–23). The reason for the greater number of recorded ACL injuries lies in the greater number of athletes, the development of diagnostics (especially MRI) (24), the greater interest of doctors in these injuries, and the development of surgical techniques and excellent postoperative results (13, 14).

Despite the large amount of research on the subject, a single standard for rehabilitation has not yet been established due to the complexity of this problem (25, 26). The goal of isokinetic testing and exercise is to provide the physiotherapist with detailed, reliable, and objective data to create an individual rehabilitation program and to gain knowledge about joint functions, bilateral imbalances, etc. Bearing in mind that isokinetic dynamometers are designed so not only that the function of a large number of different muscle groups can be assessed under controlled conditions but also that they can be used very effectively as a training tool, they have become an integral part of the restoration of muscle function in the recovery process after injuries of the locomotor apparatus, especially in monitoring rehabilitation after knee surgery. This study aimed to determine the effects of an isotonic and isometric rehabilitation program on subjects who underwent ACL surgery.

## 2 Materials and methods

We conducted a retrospective research to analyze the rehabilitation results of 180 male patients, aged 28.24 ± 4.36 years, where the first (initial, −i) isokinetic testing was performed 3–4 months (12–16 weeks) after the reconstruction of the ACL of the knee using a hamstring graft. During the sampling, the subjects were divided into two groups according to the rehabilitation protocol implemented. The examined group A-isokinetic consisted of 90 male subjects, aged 28.54 ± 4.44 years (Table 1), who underwent rehabilitation at the institute. Their rehabilitation protocol was based on isokinetic exercises five times a week to strengthen the quadriceps muscles. The examined group B-isotonic consisted of 90 male subjects, aged 27.93 ± 4.27 years (Table 1), who underwent rehabilitation at the institute, with a standard isotonic protocol for quadriceps strengthening. Before the start of the treatment, an initial isokinetic quadriceps test was performed at an angular velocity of 60 °/s. After 3–6 weeks of rehabilitation treatment, the control tests were performed following the same parameters as in the initial test. The research was conducted with the approval of the Ethics Committee of the Institute for Physical Medicine and Rehabilitation, “Dr. Miroslav Zotović” Banja Luka, (116-01-18910-1/21) dated 28/12/2021.

**Table 1:** Demographic characteristics.

Characteristic	Isokinetic group (*n* = 90)	Isotonic group (*n* = 90)
	M ± SD^a^	M ± SD^a^
Age (years)	28.54 ± 4.44	27.93 ± 4.27
Height (cm)	186.1 ± 5.03	186.51 ± 5.74
Weight (kg)	89.76 ± 8.37	90.36 ± 8.29
Body mass index (kg/m^2^)	25.91 ± 1.59	25.93 ± 1.69

^a^ Mean ± standard deviation.

### 2.1 Parameters

The isokinetic parameter of the m. quadriceps was used, i.e., maximal peak torque of the extensor (Nm) of the operated leg (EXPTRQ).

### 2.2 Experimental procedure and data analysis

In the examined group A-isokinetic (90 subjects), the subjects performed kinesitherapy according to the isokinetic exercise protocol, which consisted of a 1-day isokinetic training session lasting 45 min, with concentric/concentric contractions at multiple angular velocities. The protocol is designed so that it has a progression by days and weeks (Table 2). The isokinetic training was performed five times a week for 6 weeks. For the kinesitherapy protocol in the examined group B (isotonic exercises), a training room with a static bicycle and an EN-Dynamic apparatus used for strengthening the upper leg muscles (Enraf-Nonius B.V., The Netherlands) was used. The subjects strengthened their calf muscles using standard isotonic exercises to increase muscle strength with additional resistance. The additional resistance was progressively increased by 2%–5% BM, i.e., approximately 1–5 kg, every week, and only the number of repetitions could be increased daily during the week, not the load. The exercises on the EN-Dynamic apparatus lasted approximately 30 min (Table 2). After the end of the main part of the training, there was a short break of up to 60 s, and then stretching exercises were performed. The stretch did not last more than 5 min.

**Table 2:** Six-week exercise protocol by groups.

Exercise protocols								
Isokinetic protocol (group A)					Isotonic protocol (group B)			
Weeks	Series	Angular velocity	Number of repetitions	Pause (s)	Series	Load (% BM)	Number of repetitions	Pause (s)
I	1 1 1 2 3 3 3 3	280 °/s 240 °/s 210 °/s 180 °/s 150 °/s 120 °/s 90 °/s 60 °/s	20–25 20–22 20–22 18–20 15–18 15–18 15–18 6–8	30 30 30 30 30 30 30 30	1 2 2 3 5 5 6 1	3–5 8 12 15 20 25 30 3	20–25 18–20 15–18 12–15 10–12 8–10 6–8 20–25	60 60 120 120 120 120 120 120
II	1 1 1 3 3 4 4 4 1	280 °/s 240 °/s 210 °/s 180 °/s 150 °/s 120 °/s 90 °/s 60 °/s 280 °/s	25–30 20–25 20–25 15–20 15–18 12–15 15–18 6–8 25–30	30 30 30 30 30 30 30 30 30	1 2 2 3 5 6 7 1	5 10 15 20 25 30 35 3	20–25 15–18 12–15 10–12 8–10 6–8 5–8 20–25	60 60 120 120 120 120 120 120
III	1 1 1 3 3 4 5 5 4 1	280 °/s 240 °/s 210 °/s 180 °/s 150 °/s 120 °/s 90 °/s 60 °/s 45 °/s 280 °/s	25–30 20–25 20–25 15–20 15–18 12–15 12–15 5–8 3–5 25–30	25 25 25 25 25 25 25 25 25 25	1 2 3 4 5 7 8 1	5 10 15 20 26 31 36 3	20–25 15–18 10–12 8–10 8–10 6–8 4–7 20–25	30 30 60 60 60 60 60 60
IV	1 1 1 3 4 5 6 6 5 4 1	280 °/s 240 °/s 210 °/s 180 °/s 150 °/s 120 °/s 90 °/s 60 °/s 45 °/s 30 °/s 280 °/s	25–30 20–25 20–25 20–25 20–25 18–20 15–18 8–10 5–8 4–6 30	20 20 20 20 20 20 20 20 20 20 20	1 2 3 5 6 8 9 1	5 12 18 22 28 33 38 5	20–25 15–18 10–12 6–8 6–8 6–8 4–7 20–25	30 30 60 60 60 60 60 60
V	1 1 1 3 4 6 7 7 6 5 1	280 °/s 240 °/s 210 °/s 180 °/s 150 °/s 120 °/s 90 °/s 60 °/s 45 °/s 30 °/s 280 °/s	25–30 20–25 20–25 20–25 20–25 22–25 15–20 10–12 6–8 5–7 30	20 20 20 20 20 20 20 20 20 20 20	1 2 3 5 7 8 9 1	5 15 20 25 30 35 40 5	20–25 15–18 10–12 5–8 5–8 5–8 4–7 20–25	30 30 30 30 60 60 60 60
VI	1 1 1 3 4 6 7 7 7 6 1	280 °/s 240 °/s 210 °/s 180 °/s 150 °/s 120 °/s 90 °/s 60 °/s 45 °/s 30 °/s 280 °/s	25–30 22–25 22–25 22–25 22–25 22–25 18–22 10–15 8–10 6–8 30	20 20 20 20 20 20 20 20 20 20 20	1 2 3 5 8 9 10 1	5 15 20 25 30 35 40 5	20–25 15–18 10–12 5–8 5–8 5–8 4–7 20–25	30 30 30 30 30 30 60 60

### 2.3 Statistical analysis

Data were processed in the SPSS software (IBM Corp. Released 2010. IBM SPSS Statistics for Windows, Version 26.0. Armonk, NY, USA: IBM Corp.), and multivariate MANOVA and discriminant analysis procedures were used. Among the univariate procedures, Roy's test was applied. Data scaling was performed on contingency tables to avoid losing information, by finding the finest connections and knowledge, on non-parametric quantities. In this procedure, a real number was assigned to each class based on frequency. The fact that it is possible to apply procedures related to the scale on scaled values indicates that, in this way, new knowledge is obtained in research work, which would not have been achieved by applying procedures and methods related to non-parametric scales. Data scaling does not exclude the application of non-parametric tests. Based on the abovementioned, it can be seen that it is possible to use multivariate analysis of variance (MANOVA), discriminant analysis, and other parametric procedures and methods on scaled data. Among the univariate procedures, Roy's test, Pearson's contingency coefficient (*χ*), and multiple correlation coefficient (*R*) were applied.

By calculating the discrimination coefficient, the results of isokinetic testing that determine the specificity of the subsample and the results of isokinetic testing that were excluded from further processing, that is, the observed space was reduced, were separated. To assess the normality of the distribution of individual variables, we applied the Shapiro–Wilk test. Given that according to the above tests in all variables and time points of measurement (−i, −3, −6), the results of the entire sample did not have a normal distribution at the tested level (*p* < 0.05), we divided the entire sample into subgroups according to the sizes of the measured values, from smallest to largest: “smallest,” “smaller,” “moderate,” “larger,” and “biggest.”

## 3 Results

Table 3 presents the subgroups (classes), mean values (mean), minimum values (min.), maximum values (max.), and number of respondents with the class (*N*) in the total sample at the initial (−i) measurement, measurement after 3 weeks of rehabilitation treatment (control) (−3), and measurement after 6 weeks of rehabilitation treatment (final) (−6) for the monitored isokinetic quadriceps parameter.

**Table 3:** Maximal peak torque of the extensor (Nm) of the operated leg—EXPTRQ on tests (−i), (−3), and (−6)—grouping into classes.

EXPTRQ (Nm) classes:	Mean	Min.	Max.	*N*
Smallest	84.74	62.8	114.8	33
Smaller	154.07	119.8	169.7	64
Moderate	199.60	169.8	223.1	209
Larger	245.16	223.4	276.1	140
Biggest	304.43	276.8	330.2	94

### 3.1 Initial measurement

**Figure 1:**
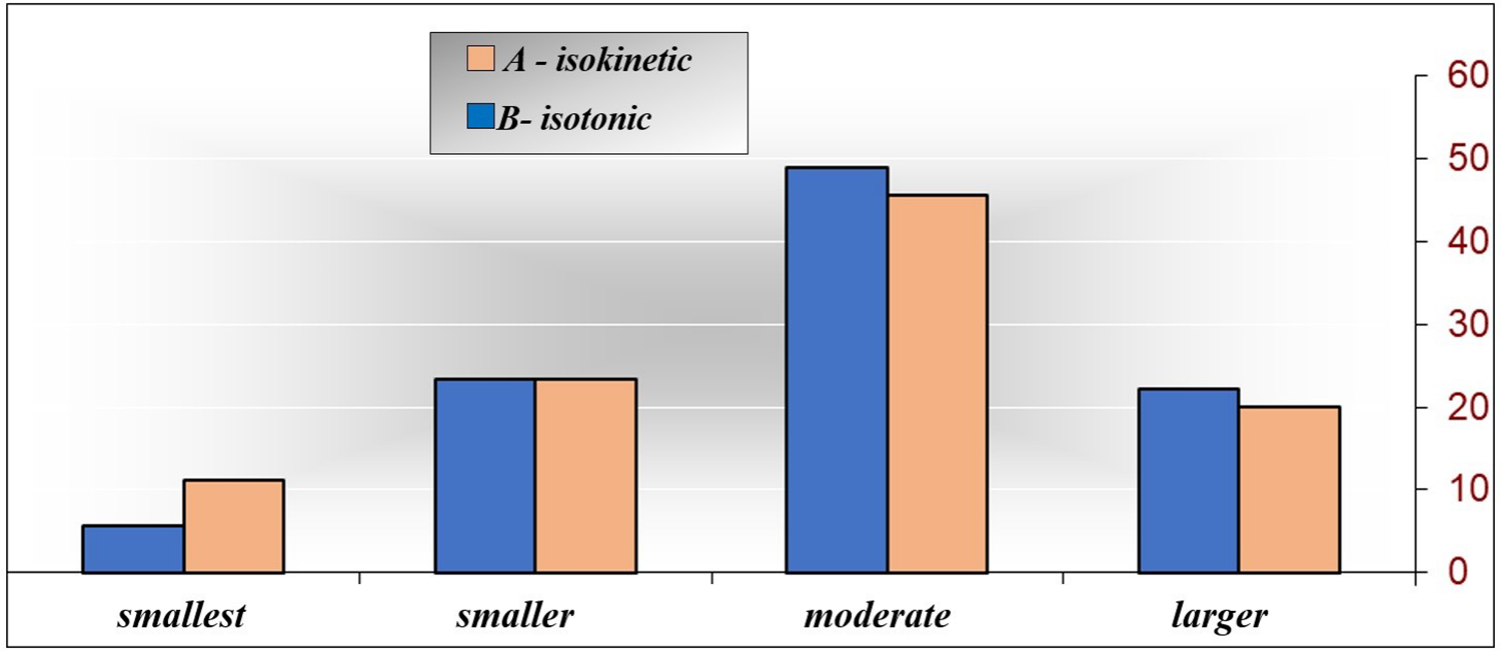
EXPTRQ classes (−i) according to the examined groups.

Table 4 and Figure 1 shows the numerical (*n*) and percentage (%) representation of classes-modalities of isokinetic parameters of extensors at the initial measurement with respect to the examined groups. Attention is drawn to significant differences, if any, between and within levels. With the descriptive procedure, it is only possible to hint at some characteristics of certain levels of isokinetic extensor parameters, while the significance of the difference between the examined groups was analyzed later.

**Table 4:** Numerical (*n*) and percentage (%) representation of classes-modality of the isokinetic parameter maximal peak torque of the extensor (Nm) of the operated leg—EXPTRQ (−i) at the initial test with respect to the examined groups.

Classes-modalities	Smallest		Smaller		Moderate		Larger	
Groups	*n*	%	*n*	%	*n*	%	*n*	%
A-isokinetic	5	5.6	21	23.3	44	48.9	20	22.2
B-isotonic	10	11.1	21	23.3	41	45.6	18	20.0

By inspecting Table 4, it is worth noting that in the examined group A-isokinetic, the most represented is the “moderate” class, comprising 44 (48.9%) out of the 90 respondents. Additionally, there is a significantly higher frequency of the “smaller” class (21 respondents, 23.3%, *p* = 0.000), followed by the “larger” class (20 respondents, 22.2%, *p* = 0.000) and the “smallest” class (5 respondents, 5.6%, *p* = 0.000). In the examined group B-isotonic, the “moderate” class is represented by 41 respondents (45.6%), which is significantly higher than the frequency of the “smaller” class (21 respondents, 23.3%, *p* = 0.002), followed by the “larger” class (18 respondents, 20.0%), (*p* = 0.000) and the “smallest” class (10 respondents 11.1% *p* = .000). The difference between the examined groups is minimal and not significant (*p* > 0.05). The “smallest” class is most represented in the examined group B-isotonic (11.11%), and the “smallest” class is most represented in the examined group A-isokinetic (23.33%). The “moderate” class is most represented in the examined group A-isokinetic (48.89%), and the“larger” class is most represented in the examined group A-isokinetic (22.22%). Based on the obtained results, it is challenging to distinguish the characteristics of each examined group regarding EXPTRQ (−i) on the initial measurement. Consequently, no characteristic was defined for the examined group A-isokinetic, while the examined group B-isotonic exhibits weak characteristics of the “smallest” class, which is of extremely weak significance. Since *p* = 0.598 of the *χ*^2^ test, it can be concluded that there is no connection between the examined groups (A-isokinetic and B-isotonic) and EXPTRQ (−i), and considering that *χ* = 0.102, the connection is very low—not significant.

### 3.2 Control measurement after 3 weeks

**Figure 2:**
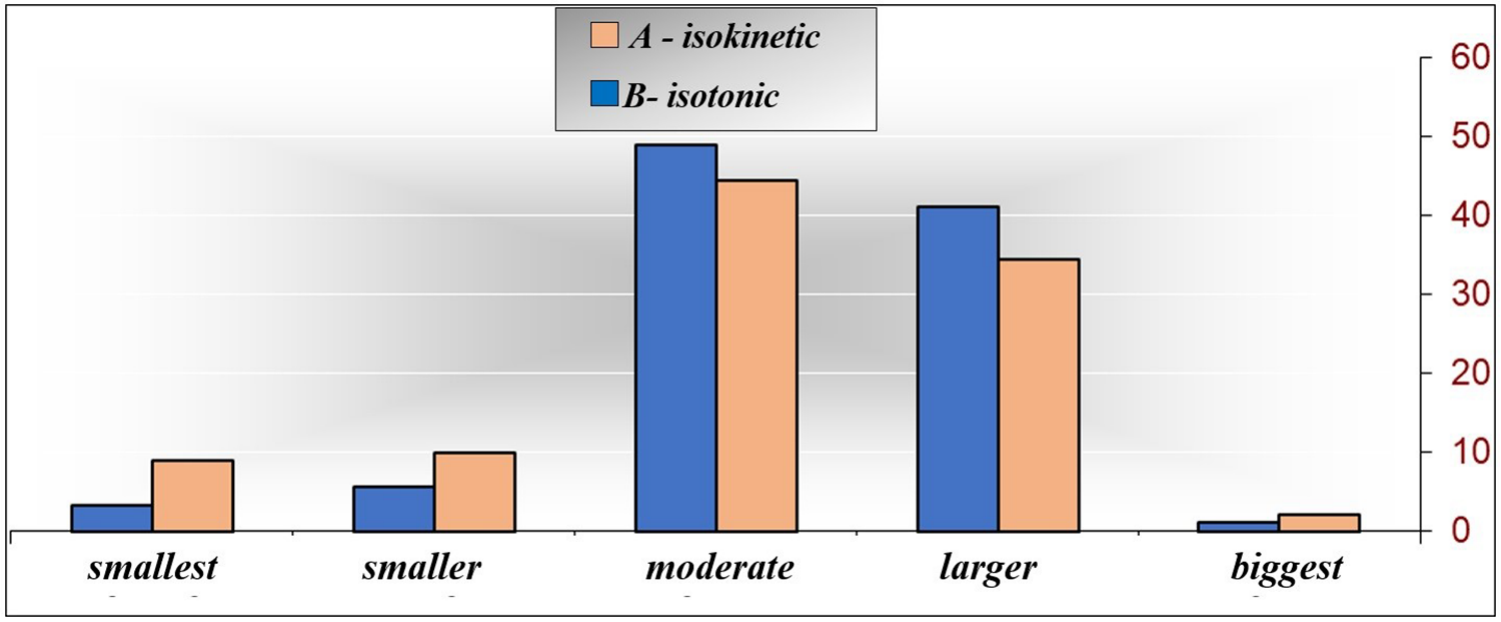
EXPTRQ (−3) classes according to the examined groups.

Table 5 and Figure 2 shows the numerical (*n*) and percentage (%) representation of the monitored isokinetic parameter. Attention was drawn to the significant differences, if they exist, between and within the levels of the examined groups. With the descriptive procedure, it is only possible to hint at some characteristics of individual levels of the isokinetic parameter, while the significance of the difference between the examined groups was analyzed later.

**Table 5:** Numerical (*n*) and percentage (%) representation of classes-modality of the isokinetic parameter maximal peak torque of the extensor (Nm) of the operated leg—EXPTRQ (−3) at the control test with respect to the examined groups.

Classes-modalities	Smallest		Smaller		Moderate		Larger		Biggest	
Groups	*n*	%	*n*	%	*n*	%	*n*	%	*n*	%
A—isokinetic	3	3.3	5	5.6	44	48.9	37	41.1	1	1.1
B—isotonic	8	8.9	9	10.0	40	44.4	31	34.4	2	2.2

By inspecting Table 5, it is possible to notice that in the examined group A-isokinetic, the most represented is the “moderate” class, comprising 44 (48.9%) out of the 90 respondents. Additionally, there is a significantly higher frequency of the “smaller” class (5 respondents, 5.6%, *p* = 0.000), followed by the “smallest” class (3 respondents, 3.3%, *p* = 0.000) and then the biggest class (1 respondent, 1.1%, *p* = 0.000). In the examined group B-isotonic, the “moderate” class is represented by 40 respondents (44.4%), which is significantly higher than the frequency of the “smaller” class (9 respondents, 10.0%, *p* = 0.000), followed by the “smallest” class (8 respondents, 8.9%, *p* = 0.000) and then the “largest” class (2 respondents, 2.2%, *p* = 0.000). The difference between the examined groups is minimal and not significant. The “smallest” class is most represented in the examined group B-isotonic (8.89%), while the “moderate” class is most represented in the examined group A-isokinetic (48.89%). The “larger” class is most represented in the examined group A-isokinetic (41.11%), while the “biggest” class is most represented in the examined group B-isotonic (2.22%). Based on the obtained results, it is very difficult to distinguish the characteristics of each examined group regarding EXPTRQ (−3) on the control measurement. Consequently, no characteristic was defined for the examined group A-isokinetic, while the examined group B-isotonic exhibits weak characteristics of the “smallest” and “smaller” classes. Since *p* = 0.346 of the *χ*^2^ test, it can be concluded that there is no connection between the examined groups and the isokinetic parameter EXPTRQ (−3), and considering that *χ* = 0.156, the connection is very low.

### 3.3 Final measurement after 6 weeks

**Figure 3:**
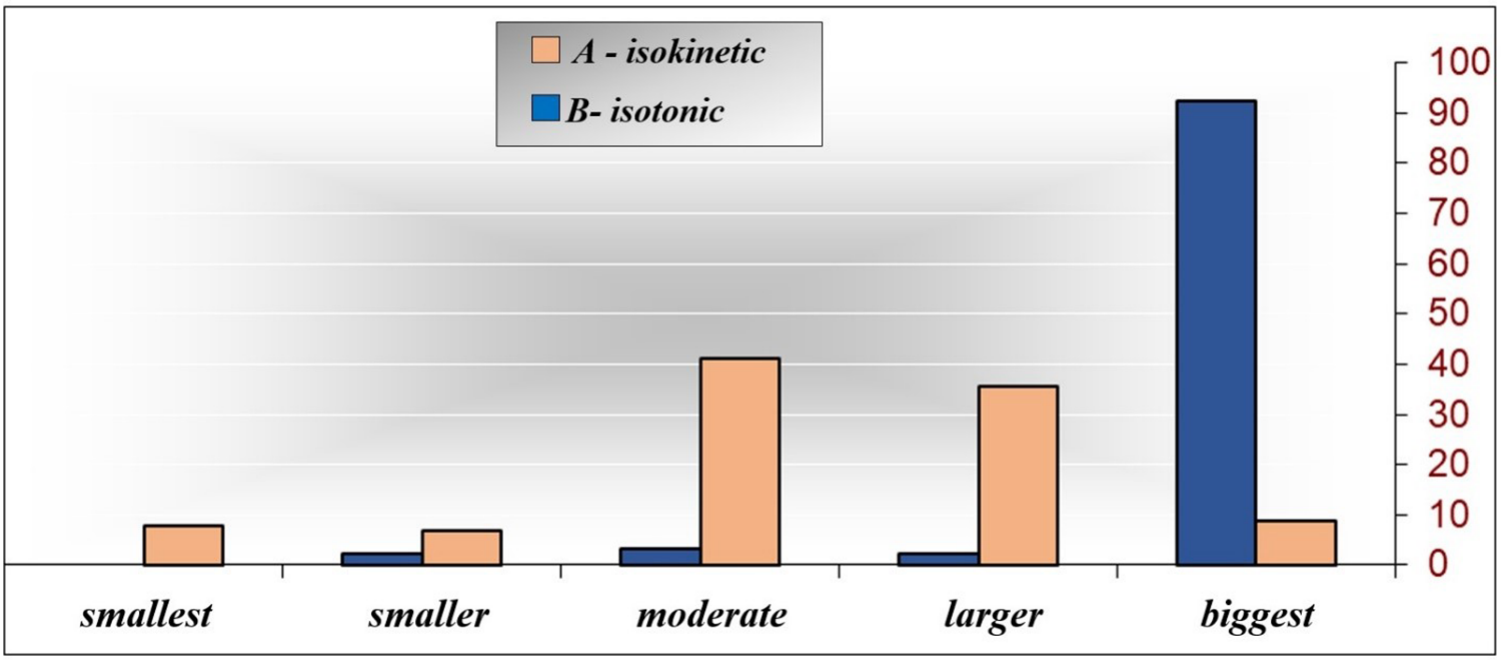
EXPTRQ classes (−6) according to the examined groups.

Table 6 and Figure 3 shows the numerical (*n*) and percentage (%) representation of the monitored isokinetic parameter at the final measurement of the examined groups. Attention is drawn to the significant differences, where they exist, between and within levels. With the descriptive procedure, it is only possible to hint at some characteristics of certain levels of the isokinetic extensor parameters at the final measurement, while the significance of the difference between the examined groups was analyzed later.

**Table 6:** Numerical (*n*) and percentage (%) representation of classes-modality of the isokinetic parameter maximal peak torque of the extensor (Nm) of the operated leg—EXPTRQ (−6) at the final test with respect to the examined groups.

Classes-modalities	Smallest		Smaller		Moderate		Larger		Biggest	
Groups	*n*	%	*n*	%	*n*	%	*n*	%	*n*	%
A-isokinetic	0	.0	2	2.2	3	3.3	2	2.2	83	92.2*
B-isotonic	7	7.8*	6	6.7	37	41.1*	32	35.6*	8	8.9

* Represents a significant difference in the results of the examined groups on the final test.

By inspecting Table 6, it is possible to notice that in the examined group A-isokinetic, the most represented is the “biggest” class, comprising 83 (92.2%) out of the 90 respondents, which is significantly higher than the frequency of the “moderate” class (3 respondents, 3.3%, *p* = 0.000), followed by the “smaller” class (2 respondents, 2.2%, *p* = 0.000), the “larger” class (2 respondents, 2.2%, *p* = 0.000), and then the “smallest” class (0 respondents, 0.0%, *p* = 0.000). In the examined group B-isotonic, the “moderate” class is represented by 37 respondents (41.1%), which is significantly higher than the frequency of the “biggest” class (8 respondents, 8.9%, *p* = 0.000), followed by the “smallest” class (7 respondents, 7.8%, *p *= 0.000) and then the “smaller” class (6 respondents, 6.7%, *p* = 0.000). The difference between the examined groups is as follows: the “smallest” class is most represented in the examined group B-isotonic (7.78%), the “smaller” class is most represented in the examined group B-isotonic (6.67%), and the “moderate” and “larger” classes are also most represented in the examined group B-isotonic with (41.11%) and (35.56%), respectively. The “biggest” class is most represented in the examined group A-isokinetic (92.22%). Based on the obtained results, it is possible to separate the characteristics of each examined group about the isokinetic parameter EXPTRQ (−6), so it follows that the examined group A-isokinetic has the most pronounced property of the “biggest” class, while the examined group B-isotonic has the most pronounced properties of the “smallest” and “moderate” classes. Since *p* = 0.000 of the *χ*^2^ test, it can be concluded that there is a connection between the examined groups and the isokinetic parameter EXPTRQ (−6), and given that *χ* = 0.642, the connection is high.

## 4 Discussion

Immediately after ACL surgery, there is hypotrophy of the m. quadriceps by 30%, which remains up to 6 months after surgery. Hypotrophy of the m. quadriceps occurs very quickly after the injury itself and is expressed in the early postoperative period after ACL reconstruction. The period of the first 3 months is very critical due to physiological changes during the healing of the graft (27). A total of 180 male patients, aged 28.24 ± 4.36 years, participated in our research, where the first isokinetic testing was performed 3–4 months (12–16 weeks) after the reconstruction of the ACL knee using a hamstring graft. From the results of our research, we see that this isokinetic parameter increases in both tested groups at the control and final measurement, with the fact that in the tested group A-isokinetic at the final measurement, this increase is significantly more pronounced. At the end of our research, analyzing the isokinetic parameter EXPTRQ, we observed the distances between the results of this parameter after 3 and 6 weeks of rehabilitation treatment. The biggest difference in the results of this parameter is between the examined group A-isokinetic at the initial and the examined group A-isokinetic at the final measurement, with a distance of 9.50, which clearly explains that isokinetic training gave convincingly better results in the increase of the torque of the anterior thigh muscle force. Subliming the results of the isokinetic parameter EXPTRQ, on all measurements, we see that there was an obvious difference according to the examined groups as the duration of the rehabilitation treatment progressed. The difference between the examined groups A and B at the initial measurement was not significant. The difference between the examined groups for this isokinetic parameter at the initial measurement was latent (0.171), so it can be said that it is not significant for our research. We can confirm that the subjects of both examined groups (A and B) had a similar “starting point,” i.e., similar possibilities for progress and further comparison of the isokinetic parameter maximum torque of the extensor force (Nm) of the operated leg—EXPTRQ. As can be seen from our results, lower extensor torque values of the operated leg prevail, which is an expected result in this period (3 months) after surgery. Lee et al. (24) observed the results of an accelerated rehabilitation protocol lasting 12 weeks and confirmed, with the help of isokinetic measurement, that there was no statistically significant recovery of the maximal knee extensor torque of the operated leg after 3 months of ACL surgery, so we see that the results of our study positively correlate with the results of the mentioned research.

At the control measurement (after 3 weeks of rehabilitation treatment), there were noticeable changes in both examined groups. An increase in values is visible in both examined groups. In the “smallest” class, the number also decreased, so in the examined group A-isokinetic, there are now three (3.33%) subjects, while in the examined group B-isotonic, there are now eight (8.89%) subjects. There is an increase in the “larger” class, so there are now 37 (41.1%) subjects in the examined group A-isokinetic, while there are now 31 (34.4%) subjects in the examined group B-isotonic. The most important thing to point out is that the first cases of the “biggest” class appear on the control measurement, so in the examined group A-isokinetic, there is now one (1.11%) subject, while in the examined group B-isotonic, there are now two (2.22%) subjects. This would certainly be a good result, but there is still a low percentage of patients who achieved EXPTRQ close to the level of a healthy leg, despite that we already see at this point that the subjects from both examined groups need continued rehabilitation treatment, especially to notice the differences between isokinetic and classical (isotonic) exercise. There is a difference among the examined groups (0.034), although looking at the classes, it seems that none of the examined groups has yet reached a significant improvement in the isokinetic parameter.

At the final measurement, i.e., the measurement after 6 weeks of rehabilitation treatment in the tested group B-isotonic in the “moderate” class, the number of subjects decreased from 40 (44.44%) to 37 (41.11%), while in the tested group A-isokinetic from 44 (48.89%), the number of subjects drastically decreased to 3 (3.33%). In the “smaller” class, there was also a decrease in both examined groups, so in the examined group B-isotonic, there are now six (6.67%) subjects, and in the examined group A-isokinetic, there are now two (2.22%) subjects. The “smallest” class was also reduced, so there are now seven (7.78%) subjects in the examined group B-isotonic, while there are no more subjects in the examined group A-isokinetic (0.0%). In the “larger” class, perhaps paradoxically, there is a drastic reduction in the examined group A-isokinetic, from 37 (41.1%), as there were in the control measurement, to only 2 (2.22%) in the final and in the examined group B-isotonic, from 31 (34.4%) to 32 (35.56%). This paradox is only clear to us when we see that in the examined group A-isokinetic class, the “biggest” class experienced a jump from 1 (1.11%) to 83 (92.22%) respondents. In the examined group B-isotonic, this shift was only from two (2.22%) to eight (8.89%) subjects. The difference between the examined groups was extremely visible and significant (*p* = 0.000). It is evident that at the end of the rehabilitation treatment in the examined group A-isokinetic, the recovery of the m. quadriceps had a much more significant increase than that in the examined group B-isotonic. It seems that it was only after 6 weeks of treatment that complete dominance of isokinetic exercise took place, that is, it took more than 3 weeks for muscle hypertrophy of the m. quadriceps. Some authors have dealt with this very problem. Matta et al. (28) confirmed the effectiveness of a 14-week application of isokinetic exercise compared with conventional exercise, to achieve selective hypertrophy of the knee extensors. The purpose of this study was to evaluate the selective hypertrophy of the m. quadriceps and increase in maximal torque of knee extensor after 14 weeks of conventional therapy compared with isokinetic therapy. The results showed uneven changes in thickness between the muscles that make up the m. quadriceps femoris. In the conventional group, there was a significantly greater thickening of the m. rectus femoris compared with all the other parts of the m. quadriceps (14%). For the isokinetic group, the increase in thickness (11%) was significantly higher compared with only the m. vastus intermedius. However, muscle thickness did not increase for all components of the m. quadriceps, relative adaptation of the m. rectus femoris, and suggested that there was a selective hypertrophy that favors this method of exercise (isokinetic) applied to the upper leg, which is in direct agreement with our research. Although the rehabilitation treatment in our research lasted much shorter, the results achieved are very close to the results in the mentioned research in terms of the maximal torque of the knee extensor. Interestingly, the similarity between the aforementioned and our research is greater in our final measurement than in the control one, which speaks in favor of the conclusion that a period of 6 weeks of rehabilitation treatment remains much more favorable for muscle function recovery of the knee extensors after reconstruction of the ACL. Furthermore, both studies confirm the dominance of the isokinetic exercise protocol compared with the isotonic one.

Another very related study (29) aimed to determine the morphological changes in one of the heads of the quadriceps muscle (m. rectus femoris) at characteristic places along the upper leg after two different training programs (isokinetic and isotonic) lasting 14 weeks. Conventional and isokinetic training caused significant increases in thickness in both places of the m. rectus femoris. While conventional training resulted in a significant increase in the cross-sectional area, isokinetic training caused a significant increase only at the distal site. The maximal torque of the knee extensor increased in the conventional and isokinetic groups, after training, regardless of the training method, although no significant changes were observed for any dependent variable in the control group. In general, the training methods resulted in similar changes in the morphology of the m. rectus femoris, while their size depended on the place on the upper leg where the measurement was made. Our research only partially positively correlates with the aforementioned, because we did not anatomically locate muscle hypertrophy. However, it has an extremely positive correlation with the aforementioned research regarding the increase in muscle performance in the examined group A-isokinetic because we also found statistically much more significant changes in that group regarding the maximum torque of the extensor force of the operated leg. It is even more interesting that the torque of the knee extensor muscle force, which we marked in our research with EXPTRQ, can be considered an index of muscle strength or, more loosely, an index of muscle progress during rehabilitation. The group of authors also proved the dominance of isokinetic exercise that was carried out as part of the rehabilitation treatment, and the result was a statistically significant improvement in the torque value of the extensor force at an angular speed of 60 °/s (30), which fully corresponds to the method and the results of our research.

The analysis of only the maximum torque of the extensor force, that is, only one isokinetic parameter, has certain limitations. Also, some studies highlight the importance of performance reporting m. quadriceps during certain flexion angles during the entire range of motion of the knee, identifying possible irregularities or asymmetries of muscle performance (31). To evaluate the results in the aforementioned research, the authors use an isokinetic test angular velocity of 60 °/s, which positively correlates with our research.

The limit of our research is that the respondents had different occupations and different levels of activity indicating that they come from diverse populations. Consequently, we could not expect normally distributed values in such a large sample. Since the study was conducted retrospectively, we believe that the results obtained from such a large sample will be valuable to everyone involved in the rehabilitation and recovery of individuals who underwent ACL surgery.

## 5 Conclusion

There is a significant difference in the results of the isokinetic parameter EXPTRQ between the examined groups at the final measurement. It is evident that at the end of the rehabilitation treatment in the examined isokinetic group, recovery of the m. quadriceps, i.e., isokinetic parameter EXPTRQ had a much more significant increase than in the studied isotonic group. It seems that complete dominance of isokinetic exercise occurred only after 6 weeks, that is, it took more than 3 weeks for muscle hypertrophy to occur in the m. quadriceps. We see that isokinetic exercise is a more successful method for achieving quadriceps muscle strength in patients after ACL reconstruction than isotonic exercise. In future research, it would be good to analyze all isokinetic parameters, first of all, the maximum torque of muscle force of both legs, muscle strength, and the ratio of agonists and antagonists, and compare their progress or decrease on control measurements. We believe that following the results of this research, it would be worthwhile to consider investigating the impact of isokinetic exercise on the prevention of ACL injuries, as well as examining how preinjury isokinetic exercises affect the outcome of rehabilitation post-injury. Additionally, exploring whether the ACL prevents the early development of knee arthrosis could be an area of interest for future research. Given the contradictory findings in previous research, this topic holds potential for further investigation.

## Data Availability

The original contributions presented in the study are included in the article/Supplementary Material, further inquiries can be directed to the corresponding author.
